# Correlation of opportunistic bone mineral density assessment derived from CT images with physical functioning in long-term follow-up in patients with rib fractures

**DOI:** 10.3389/fsurg.2026.1911891

**Published:** 2026-10-23

**Authors:** Benedikt Heyart, Daniele Romeo, Ulrich Spiegl, Christian Kleber, Sebastian Krämer, Timm Denecke, Hans-Jonas Meyer

**Affiliations:** 1 Department of Diagnostic and Interventional Radiology, University Hospital Leipzig, Leipzig, Germany; 2 Department of Biomedicine, Neuroscience and Advanced Diagnostics (BiND), University of Palermo, Palermo, Italy; 3 Department of Trauma Surgery and Orthopaedics, München Klinik Harlaching, Munich, Germany; 4 Department of Orthopaedics, Trauma Surgery and Plastic Surgery, University Hospital Leipzig, Leipzig, Germany; 5 Department of Visceral, Transplant, Thoracic and Vascular Surgery, Division of Thoracic Surgery, University Hospital Leipzig, Leipzig, Germany

**Keywords:** bone mineral density, CT, health-related quality of life, physical functioning, rib fracture, SF-36

## Abstract

**Objective:**

Quality of life assessment is strictly related to patients physical functioning. Despite the fact that earlier research has concentrated on the role of osteoporosis, the question of whether opportunistic bone mineral density (BMD) assessment can assist in determining physical functioning remains unresolved. This exploratory analysis aims to elucidate whether the opportunistic BMD assessment defined by computed tomography (CT) images is associated with quality of life in long-term follow-up in patients with traumatic rib fractures.

**Methods:**

All patients had rib fractures without polytrauma. A total of 72 patients, including 27 female patients (37.5%), with a median age of 60 years, were included in the present study. Contrast-enhanced CT imaging was obtained and vertebral trabecular attenuation in HU at Th12 level was measured. CT scans were perfomed using a tube voltage of 120 kVp. Quality of life was assessed using the Short Form 36-Item Health Survey (SF-36) no earlier than four years after trauma.

**Results:**

Opportunistically measured BMD obtained at 120 kVp showed a moderate correlation with the physical functioning (PF) with a *r* = 0.43 (*p* ≤ 0.001). The severely impaired PF group showed a significantly lower level of vertebral trabecular attenuation with a median value of 118 HU compared to 172 HU of the non-impaired group (*p* = 0.004). CT-derived BMD showed a moderate predictive ability for severely impaired PF (AUC = 0.78) with a cut-off level of 132 HU.

**Conclusion:**

CT-derived opportunistic BMD measurement at Th12 level is correlated with severe physical functioning impairment in patients with rib fractures in long-term follow-up. The potential role of CT images in patients with traumatic rib fractures, beyond diagnostic purposes, needs further assessment.

## Introduction

In recent decades, there has been a marked shift in the focus of medical research from biomechanical endpoints, such as mortality, to a greater emphasis on the assessment of long-term quality of life (1, 2), for example in trauma patients (3–5).

The WHO defines quality of life as “an individual's perception of their position in life in the context of the culture and value systems in which they live and in relation to their goals, expectations, standards and concerns.” (6).

Health-related quality of life (HRQoL) is usually understood in the literature as the health-related part of quality of life as defined by the WHO (7). A range of measurement tools has been developed to operationalize this multifactorial concept of quality of life, thereby enabling its quantification and comparison.

A notable instrument that has gained international recognition and is frequently utilized in research is the SF-36 (Short Form 36) questionnaire (8). Measuring eight health items (physical functioning (PF), role limitations due to physical problems (RP), bodily pain (BP), general health (GH), vitality (VT), social functioning (SF), role limitations due to emotional problems (RE), mental health (MH)), which define a physical and mental dimension (physical component summary (PCS), mental component summary (MCS), the SF-36 has been extensively utilized and adapted to the norm population of various countries (9–11). Therefore the SF-36 has often been used to identify risk factors influencing the HRQoL both for the general population (9) and in different patient cohorts (12, 13). Quality of life indicators can be used not only to evaluate therapies, but also to plan them to prevent reduced mobility and high healthcare costs (14).

Besides demographic factors (15–17) and disease-related factors such as fracture severity in trauma patients (18–20) biomedical factors such as osteoporosis have been evaluated (21). In recent years, there has been a notable increase in the focus on bone mineral density (BMD), a quantitative assessment of bone mineralization, as a metric for the quantification of osteopenia and osteoporosis (22, 23). Previous studies examining the influence of BMD on HRQoL have utilized the dual-energy x-ray absorptiometry (DXA) to measure BMD (24–26). An alternative approach used in literature for BMD assessment, highly correlated with DXA-derived BMD, involves the opportunistic measurement of trabecular bone attenuation in Hounsfield units (HU) within vertebral bodies, from pre-existing computed tomography (CT) scans (27, 28). This method is cost-efficient, does not require additional radiation exposure, and can be analyzed in an automated fashion. This is especially advantageous in trauma patients, which are likely to undergo CT scans for the purpose of trauma evaluation during clinical routine.

Therefore, the objective of this study was twofold: first, to evaluate the influence of opportunistic CT-derived BMD in trauma patients without polytrauma on long-term HRQoL; and second, to compare the results with the existing literature regarding DXA-measured BMD.

## Methods

### Ethical consideration

This retrospective study was approved by the institutional ethic committee (internal reference number: No. 420/20-ek) and informed consent was waived due to the study design. The study was performed in accordance with the ethical standards of the institutional and/or national research committee and with the 1964 Helsinki declaration and its later amendments or comparable ethical standards.

### Patient sample

All patients of this study were treated for rib fractures between January 2014 and December 2018 at a Level I trauma center. Inclusion criteria for this study were: age greater than 18 years, Injury Severity Score (ISS) less than or equal to 16, a minimum follow-up of 4 years; presence of contrast media enhanced CT with evaluable Th12 mineral density and conservative therapy of the fractures. Patients with an Abbreviated Injury Scale (AIS) of 4 and therefore an ISS of 16 were only included if they suffered a thoracic monotrauma, so that the definition of polytrauma did not apply. Exclusion criteria were: pregnancy, dementia, mental illness or physical disability, cancer, spontaneous fractures, suicidal tendencies and not assessable images due to image quality or artifact.

Patient information was extracted from the internal database. Study patients also provided information on health-related quality of life, current pain and respiratory function using the German version of the 36-item Short-Form Health Survey (SF-36) and a visual analog scale (VAS) with a minimum of 4 years after trauma (29).

### SF-36 assessment

The HRQoL was assessed using the German version of the SF-36 Questionnaire. The measurement involved the quantification of eight health items: physical functioning (PF), role limitations due to physical problems (RP), bodily pain (BP), general health (GH), vitality (VT), social functioning (SF), role limitations due to emotional problems (RE) and mental health (MH), and therefore calculates and defines a physical and mental dimension (physical component summary (PCS), mental component summary (MCS) using the eight abovementioned domains (8). The overall score ranges from 0 to 100 with a higher score defining a more favorable health state.

### CT imaging

Contrast-enhanced CT was performed in a clinical setting using a 128-slice CT scanner (Ingenuity 128, Philips, Hamburg, Germany). Iodine-based contrast medium (90 mL Imeron® 400 MCT, Bracco Imaging Germany GmbH, Konstanz, Germany) was administered intravenously at a rate of 2–4.0 mL/s. Automatic bolus tracking was performed in the descending aorta with a trigger of 100 Hounsfield units (HU). CT images were obtained in the late arterial phase in every case. Typical imaging parameters were as follows: 120 kVp; 125 mAs; slice thickness, 1 mm; and pitch, 0.9. Digital Imaging and Communications in Medicine (DICOM) data were retrieved from the institutional Picture Archiving and Communication System (PACS) for volumetric analysis.

### Bone mineral density assessment

The vertebral trabecular attenuation in HU was measured at the level of the 12th thoracic vertebrae. A circular region of interest (ROI) was manually drawn including an area corresponding to the anterior part of the vertebral body on the selected slice, avoiding the compact bone of the epiphyseal ring and the venous plexus, and the average CT scan density of the ROI was measured (30). CT images were analyzed within the clinical used PACS by a trained resident with 3 years of experience.

A subset of 23 patients (32% of all patients) was analyzed by another reader with 3 years of general experience to provide an interreader agreement for the measurement.

A representative patient of the cohort is displayed in Figure 1 for illustrative purposes.

**Figure 1:**
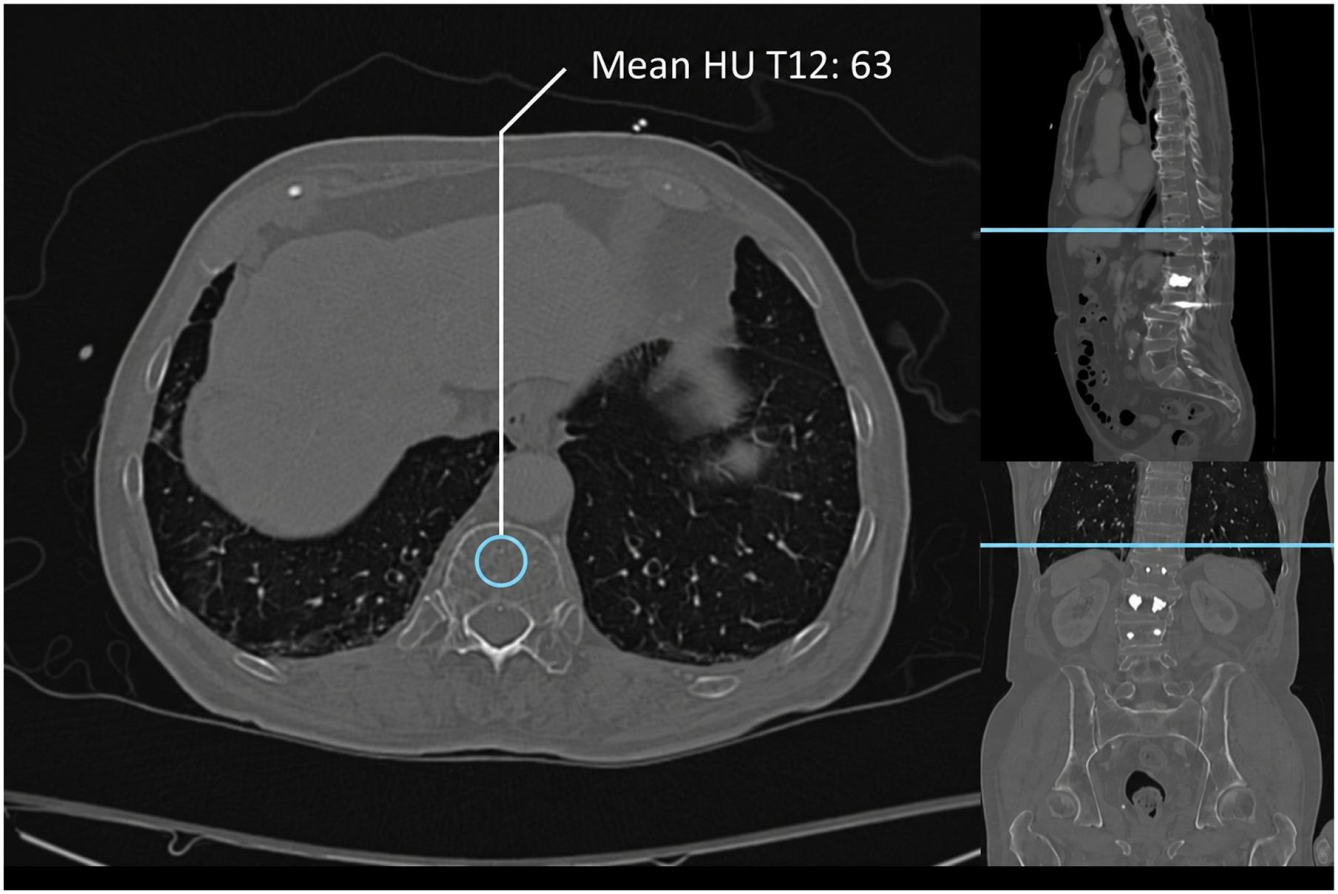
Representative opportunistic CT-derived BMD measurement of a male patient with age >60 years old at the level of the 12th thoracic vertebrae. The measurement was performed in contrast-enhanced CT during trauma evaluation. The region of interest was placed in the anterior part of the vertebral body at Th12 level. No compact bone or artifacts were included in the ROI. The resulting opportunistic measurement of this patient is 63 HU.

### Statistical analysis

All statistical analyses were performed using RStudio (Version 2024.12.1, R 4.4.3., http://www.r-project.org/, R Foundation for Statistical Computing). Continuous variables are presented as medians with interquartile ranges (IQR) and categorical variables as absolute and relative frequencies. Mann Whitney-U test and Fisher's exact test were used to perform comparison between groups, when suitable. The interreader agreement for the BMD measurement was analyzed with intraclass coefficients (ICC). Diagnostic accuracy was tested with receiver Operating Characteristic (ROC) analysis using the area under the curve (AUC) as outcome parameter. Youden index was performed to set the cut-off. Spearman's correlation was used to assess the association between BMD with PF and BMD with BMI. Physical functioning was dichotomized at a value of 40 to indicate severe physical impairment. The exploratory cutoff was defined as one standard deviation below the mean of the population's PF and aligns with previously explored cut-off in the literature (31). In a similar manner, the variable of age was dichotomized at 60 years (< 60 vs. ≥ 60 years), which reflects the median value of the study population and aligns with established practices in related research, which has defined 60 years as a cutoff for elderly individuals (32). The BMI cut-off was established at 25 kg/m^2^, as this value has been shown to correlate with an elevated risk of cardiovascular diseases and metabolic syndrome, which can potentially compromise quality of life (33, 34). In all instances, tests were two-sided and *p* < 0.05 was considered statistically significant.

## Results

Altogether, 72 patients (27 women (37.5%) with a median age of 60 (IQR: 49-71) met the inclusion criteria and were included into the present study. The general population characteristics are shown in Table 1.

**Table 1:** Characteristics of the population included in the study.

Variable	N (%) or Mdn (IQR)
Sex (females)	27 (37.5%)
Age	60 (49–71)
BMD Th12 (HU)	157 (120.5–196)
BMI	26.23 (23.86–28.96)
Follow-up Time (years)	5 (4–6)
Number of Fractures	3 (1–5)
Physical functioning (PF)	82.5 (53.75–100)
Role physical (RP)	100 (25–100)
Bodily pain (BP)	62 (41–100)
General health (GH)	62.2 (41.65–72.2)
Physical component summary (PCS)	46.23 (36.76–54.8)
Vitality (VT)	60 (40–75)
Social Functioning (SF)	87.5 (50–100)
Role emotional (RE)	100 (33.33–100)
Mental health (MH)	72 (52–84)
Mental component summary (MCS)	51 (40.09–56)
Current pain	1 (1–4)
Current respiratory problems	1 (1–4)
Lower Limbs injury	11 (15.3%)
Upper Limbs Injury	19 (26.4%)
Trunk Injury	41 (56.9%)
Head Injury	19 (26.4%)

Scores for “Current pain” and “Current respiratory problems” were queried by visual analog scale (scale range, 1 to 10) and are dimensionless. The PCS and MCS values were calculated from the subjects’ responses to the Short Form 36 (SF-36) health status questionnaire and are dimensionless.

The median follow-up time was 5 years. Among these, 13 patients showed severe physical impairment, while 59 had physical functioning ≥ 40. The median BMD at the 12th thoracic vertebra was 157 HU (IQR: 120.5-196).

### Inter-reader variability in BMD assessment

Two readers independently evaluated the CT-derived BMD at Th12 level. The ICC was 0.88 (95% CI = 0.74–0.95) showing an excellent agreement between the two independent observers.

### Discrimination analysis

Discrimination analysis was conducted between the groups of patients with and without severe physical impairment. In the group with severe physical impairment (physical functioning < 40), role physical (100 vs. 12.5, *p*-value < 0.001), bodily pain (77 vs. 31, *p*-value < 0.001), physical component summary (49.1 vs. 22.3, *p*-value < 0.001), vitality, social functioning, role emotional and mental component summary, were significantly lower, indicating a general deterioration of physical condition and social function among patients. Mental health (MH) related parameter showed a tendency to lower values in group with severely impaired PF.

Patients exhibiting physical functioning < 40 demonstrated a significant decrease in median vertebral trabecular attenuation (118 HU vs. 172 HU, *p*-value = 0.004). Moreover, this subgroup exhibited a comparatively higher proportion of patients aged > 60 years (84.6% vs. 40.7%, *p*-value = 0.005).

The results of the discrimination analysis are presented in Table 2.

**Table 2:** Comparisons between the two physical functioning groups.

	Physical functioning ≥ 40		Physical functioning < 40		
Variable	*Mdn* or *n*	*IQR* or %	*Mdn* or *n*	*IQR* or %	*p*
Sex (female)	19	32.2	8	61.5	0.062
Age > 60	24	40.7	11	84.6	0.005
BMI > 25	27	57.4	10	83.3	0.179
BMD Th12 (HU)	172	131.5–198.5	118	98.2–135.2	0.004
Lower Limbs injury	10	16.9	1	7.7	0.676
Upper Limbs Injury	17	28.8	2	15.4	0.491
Trunk Injury	33	55.9	8	61.5	0.767
Head Injury	18	30.5	1	7.7	0.162
Number of Fractures	3	1;5	4	2–5.5	0.326
Rib Series Fracture	27	45.8	8	61.5	0.367
Physical functioning (PF)	90	70–100	20	15–30	<0.001
Role physical (RP)	100	50–100	12.5	0–25	<0.001
Bodily pain (BP)	77	61–100	31	21–52	<0.001
General health (GH)	67.2	52.2–77	40	30–45	<0.001
Physical component summary (PCS)	49.1	42.6–55	28.2	22.6–31	<0.001
Vitality (VT)	65	47.5–75	40	20–50	<0.001
Social Functioning (SF)	87.5	50–100	50	37.5–62.5	0.008
Role emotional (RE)	100	100–100	0	0–100	0.002
Mental health (MH)	75	56–84	52	48–76	0.057
Mental component summary (MCS)	51	41.8–56.8	49.8	35–53	0.178
Current Pain	1	1;3	5	3;5	<0.001
Current respiratory problems	1	1;2	4	3;2	0.001

Scores for “Current pain” and “Current respiratory problems” were queried by visual analog scale (scale range, 1 to 10) and are dimensionless. The PCS and MCS values were calculated from the subjects’ responses to the Short Form 36 (SF-36) health status questionnaire and are dimensionless.

### Diagnostic accuracy and BMD cut-off determination

The ROC analysis showed a high accuracy for the CT-derived BMD to predict impaired physical functioning (PF < 40) with an AUC of 0.78. The best cut-off value was identified at 132 HU. The corresponding graph is shown in Figure 2.

**Figure 2:**
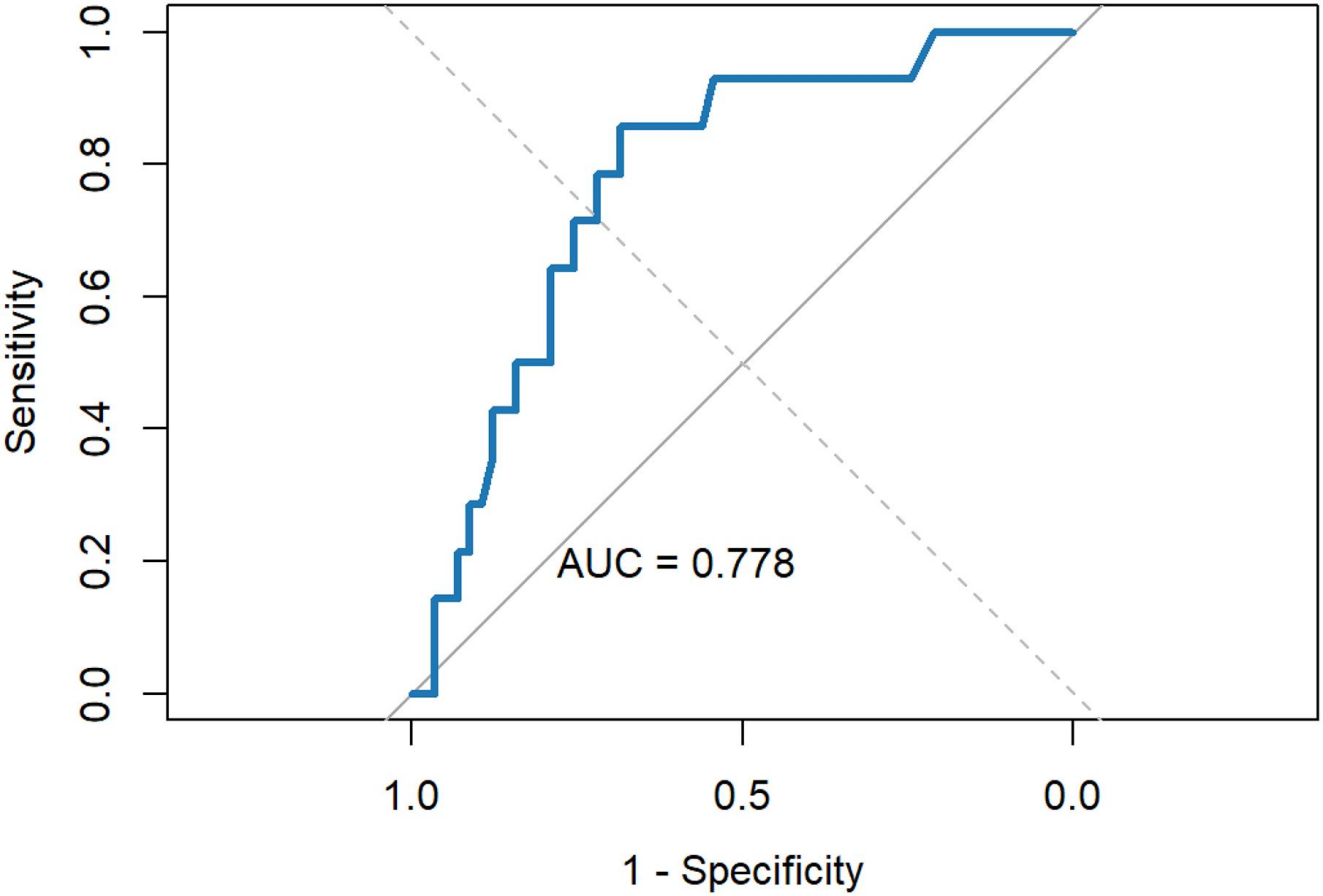
ROC curve showing the diagnostic accuracy of BMD (HU) to predict physical functioning. The resulting area under the curve is 0.78 using a cut-off value of 132 HU.

### Comparison between low and high BMD groups

Fisher's exact test was used to perform the comparison according to BMD values. The group with a CT-derived BMD value lower than 132 HU was composed of 24 patients (33.3% of all patients). Among them, 9 (37.5%) showed severely impaired physical functioning. Only 3 patients (6.4%) showed a PF < 40 in the group with higher BMD (*p*-value = 0.002). This result is shown in the stacked bar plot in Figure 3.

**Figure 3:**
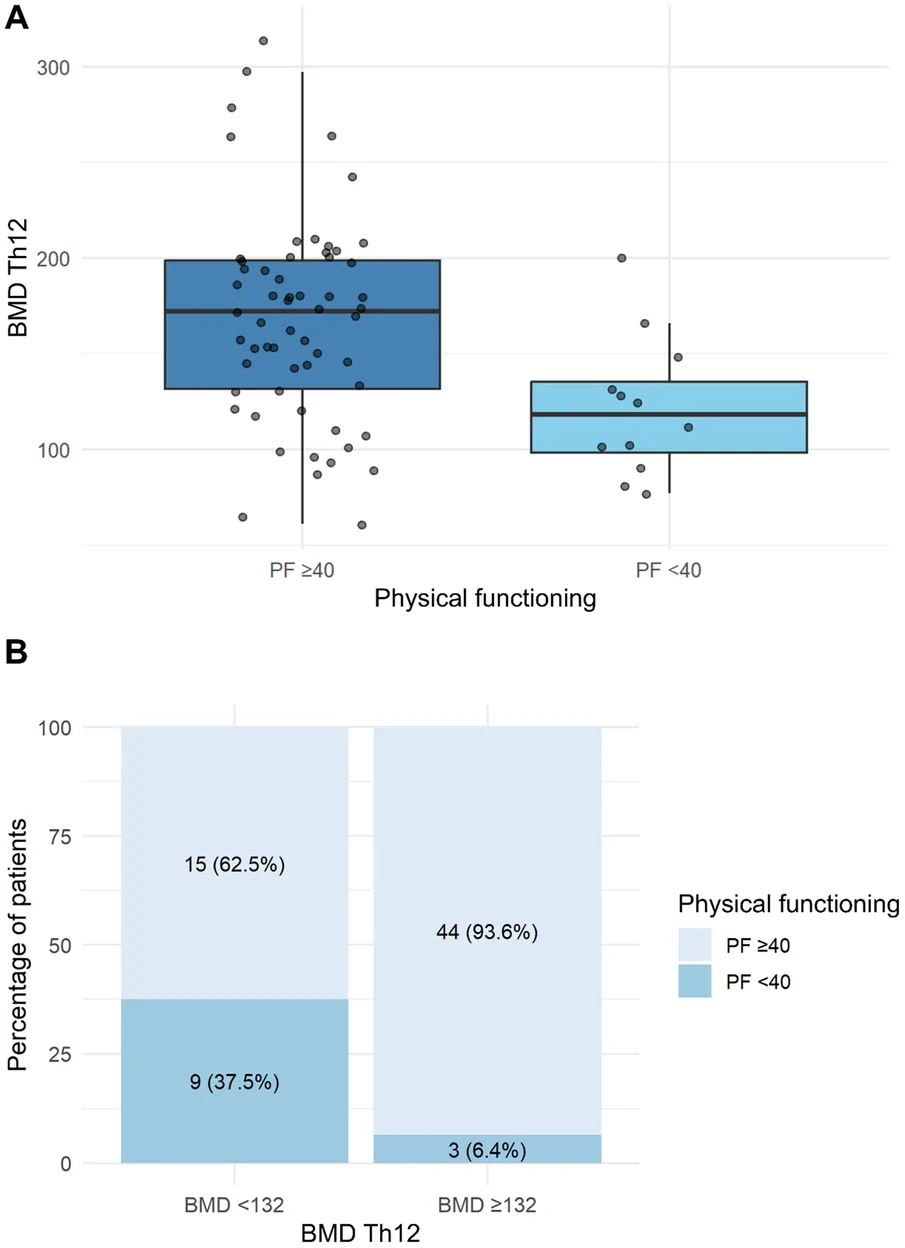
**A.** Box Plot showing the different BMD values in patients with low and high physical functioning (Mann–Whitney-U test, *p* = 0.004). **B.** Stacked Bar Plot showing the different prevalence of low and high physical functioning according to low and high BMD values (Fisher's exact test, *p* = 0.002).

### Correlation analysis between BMD and physical functioning

CT-derived BMD and PF showed a positive moderate correlation with *r* = 0.43 (*p* ≤ 0.001).

The correlation graph between these parameters is shown in Figure 4. Other parameters as physical component summary (PCS), social functioning (SF) and general health (GH) showed only weak correlations with the opportunistic BMD assessment, *r* = 0.29 (*p* = 0.015), *r* = 0.29 (*p* = 0.016) and *r* = 0.25 (*p* = 0.035), respectively.

**Figure 4:**
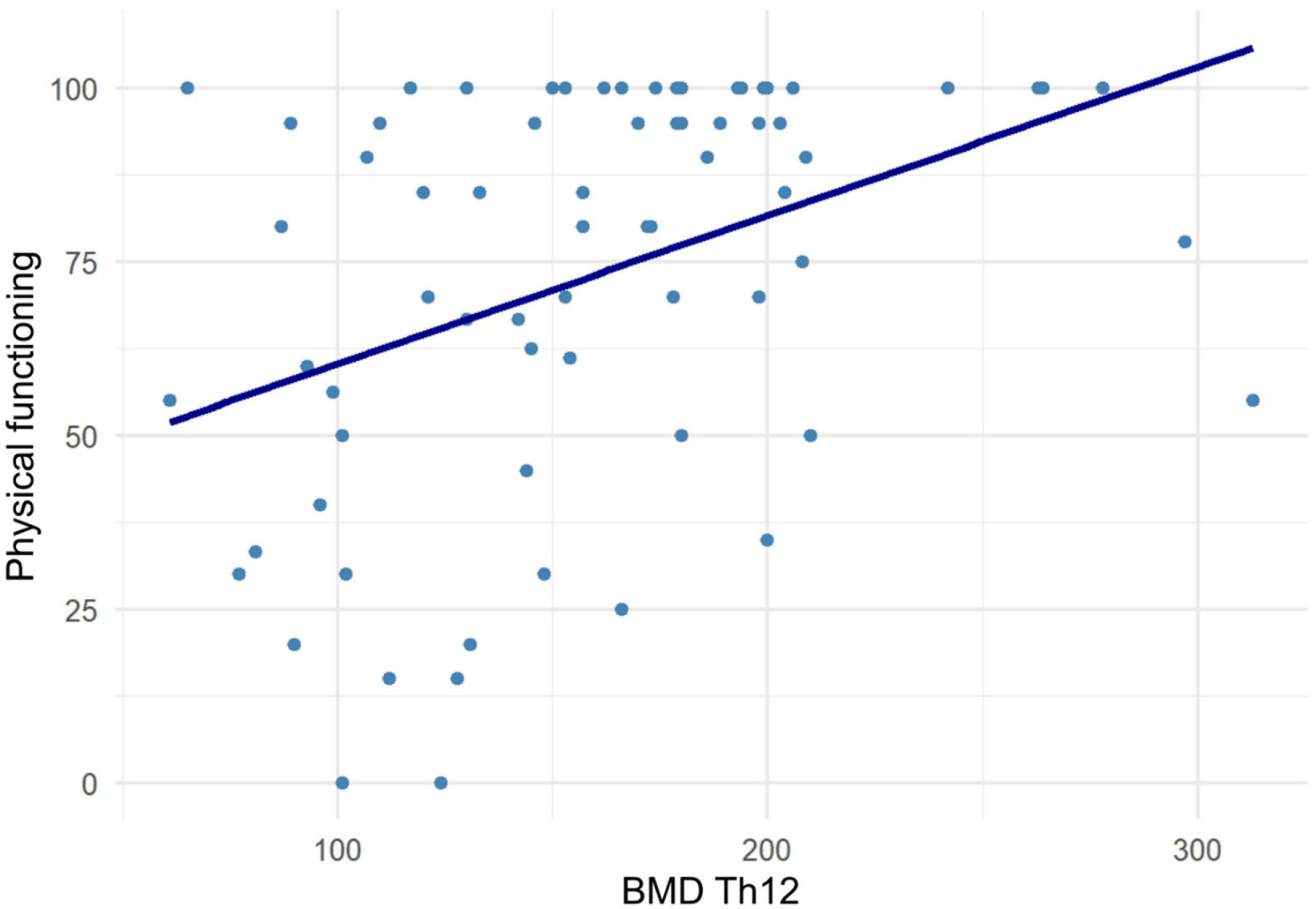
Spearman's correlation analysis between bone mineral density and physical functioning (r = 0.43, *p* < 0.001).

All the other SF-36 assessed parameters, such as vitality (VT), mental health (MH), role physical (RP), role emotional (RE), mental component summary (MCS) and bodily pain (BP) showed no significant correlation with *p* > 0.05.

## Discussion

Opportunistic BMD assessment via CT images can accurately assess the bone structure of healthy and osteoporotic bone (35). Although DXA still remains the gold standard for bone assessment, CT opportunistic measurement of trabecular bone attenuation showed good to high correlations with DXA itself (35). It also constitutes a cost-effective widely available quantitative index, able to provide information about prognosis in clinical, surgical and oncological conditions (36–38). However, the majority of studies have focused on the femur and the lumbar spine for BMD measurement (24, 26, 39).

Opportunistic CT-derived trabecular bone attenuation measurement, however, is not the only way to assess bone health and risk fracture in clinical practice. Trabecular bone score (TBS), a texture-based index that reflects bone microarchitecture, has demonstrated to improve fracture risk prediction and has been successfully integrated into Fracture Risk Assessment Tool (FRAX) (40). However, according to ISCD recommendation, TBS should not be used alone to guide patients’ treatment but be used as a complementary tool to refine the fracture risk prediction. Therefore, the exploration of new predictive tools that may improve clinical outcomes and risk prediction is warranted. Our opportunistic assessment, in particular, was performed on the Th12 level, routinely included in CT scan for rib fracture assessment, requiring no additional examinations or radiation exposure.

In the field of literature, a decline in DXA-measured BMD was found to be associated with a higher likelihood of experiencing poor HRQoL. This is particularly evident in the male population, as a low BMD has been shown to have more significant consequences in males than in females (21, 24, 25, 39). In the context of the SF-36 survey, Dennison et al. identified the physical functioning subscale as being of the utmost importance (26). These findings align with the conclusions drawn from our study, demonstrating a positive correlation between trabecular bone attenuation and HRQoL with a significant emphasis on the physical functioning (PF) (*r* = 0.43, *p* *≤* *0.001)*. When used as predictor, the opportunistic assessment showed a moderate accuracy in prediction of low PF with an AUC of 0.78. A cut-off of 132 HU was therefore determined. Interestingly, this cut-off aligns with the previous found cut-off for osteopenic bone showing consistency with previous literature (41, 42).

The patients’ sub-group with low vertebral trabecular attenuation levels showed a major prevalence of severely impaired physical functioning in the follow-up (37.5% vs. 6.4%, *p* = 0.002). Comparison analysis between patients with and without severe PF impairment, demonstrated higher levels of acute pain, respiratory problems and lower values of other physical subscales, confirmation of the high factorial validity and reliability of the SF-36 (9). The severity of trauma, as indicated by the number of fractures, was not found to be a significant factor. This finding aligns with the conclusions of other studies, which have demonstrated that the ISS does not possess any prognostic significance, even in patients with polytrauma (43–45). In previous studies, has been shown that older age (16, 17, 46) and higher BMI (15, 47) are associated with poorer physical quality of life. Furthermore, several risk factors are recognized to be associated with reduced BMD and increased fracture risk, such as sex, menopausal status, comorbidities and physiological conditions affecting calcium metabolism, such as pregnancy and lactation, as well as metabolic pathological conditions affecting bone health, potentially representing confounding factors (48–50).

In our analysis, patients with a higher age were more represented in the cohort with severe PF impairment, no differences about BMI were observed in the present cohort (*p* > 0.05), and no correlation between CT-derived BMD and BMI was detected. However, it needs to be accounted that the population object of the present analysis shows a median BMI of 26.23 kg/m^2^, potentially influencing the ability of detecting a correlation between BMI and BMD itself. Interestingly, presence of other lesions in other body districts, did not show significant differences among the groups with high and impaired PF (all *p*-values > 0.05). This sustains our hypothesis that opportunistic CT-derived BMD assessment could reflect the more complex and delicate metabolic balance (51), showing its effect mainly in the medium- and long-term follow-up of trauma patients in terms of PF and quality of life, while the number of lesions could be more prognostic in the short term (52). We speculate that the worse PF in the patients’ minimum 4-years follow-up, could reflect the general health condition prior to the trauma and, therefore, a higher risk of lower HRQoL in patients with traumatic rib fracture. This could therefore constitute a first step in bone health assessment, potentially identifying patients with an increased risk of impaired PF and risk fracture. These patients could, in fact, benefit from an early assessment using DXA or FRAX, and, if clinically indicated, from the early implementation of preventive or therapeutic strategies.

The present analysis has several limitations to address. It is a retrospective, single-center study with a relatively small cohort, prone to selection bias. Furthermore, the small cohort could hinder the statistical power of the study. Therefore, the nature of the study can be considered as explorative. The two cohorts differ significantly in median age, underscoring the necessity for additional, more extensive, and comprehensive studies incorporating multivariate analysis to elucidate the role of BMD among potential strong confounders. Furthermore, sufficiently detailed data regarding menopausal status in female patients, previous fragility fracture and disorders affecting bone metabolism were not present, further underscoring the need of external validation and a more comprehensive risk stratification in a larger population. No correlation between BMI and BMD was observed in the present study. The relatively high BMI of the population may have partially influenced this finding and should be considered among the limitations of the present analysis.

No phantom calibration was performed, nor DXA was available for the included patients. Therefore, no comparison to the current gold standard of bone mineral assessment can be performed in our study. Although this should need to be addressed in the limitations, our study is in line with the current knowledge, cut-offs, and literature regarding BMD and quality of life indicators. Furthermore, the present analysis was performed on contrast-enhanced CT images, because of the available clinical images to rule out traumatic findings. Contrast media could influence the HU measurement within the vertebral body (27). Although the measurement was performed in the anterior part of the vertebra to partially address this issue, this aspect should be reported as unavoidable limitation of the study design.

In conclusion, CT-based BMD is associated with severe physical functioning impairment in patients with rib fractures, in a 4-year follow-up. Prospective evaluation of CT-derived BMD in patients who sustain rib fracture could constitute a first opportunistic step in bone health assessment. This could potentially identify patients who may benefit from a more targeted bone health assessment and, if clinically appropriate, preventive or therapeutic strategies.

## Data Availability

The raw data supporting the conclusions of this article will be made available by the authors, without undue reservation.
